# Inhibition of DYRK1B suppresses inflammation in allergic contact dermatitis model and Th1/Th17 immune response

**DOI:** 10.1038/s41598-023-34211-x

**Published:** 2023-04-29

**Authors:** Thamrong Wongchang, Panwadee Pluangnooch, Suradej Hongeng, Adisak Wongkajornsilp, Dean Thumkeo, Kitipong Soontrapa

**Affiliations:** 1 Department of Pharmacology, Faculty of Medicine Siriraj Hospital, Mahidol University, 2 Wanglang Road, Bangkoknoi, Bangkok, 10700 Thailand; 2 Division of Pharmacology, Department of Pharmaceutical Care, School of Pharmaceutical Sciences, University of Phayao, Phayao, Thailand; 3 Department of Pediatrics, Faculty of Medicine Ramathibodi Hospital, Mahidol University, Bangkok, Thailand; 4 Excellent Center for Drug Discovery, Mahidol University, Bangkok, Thailand; 5 Department of Drug Discovery Medicine, Medical Innovation Center, Kyoto University Graduate School of Medicine, Kyoto, Japan

**Keywords:** Drug discovery, Immunology

## Abstract

Allergic contact dermatitis (ACD) is a type IV hypersensitivity mainly mediated by Th1/Th17 immune response. Topical corticosteroid is currently the first-line treatment for allergic contact dermatitis (ACD) and systemic administration of immunosuppressive drugs are used in patients with severe disseminated cases. However, increased risk of adverse effects has limited their use. Thus, the development of a novel immunosuppressant for ACD with low toxicity is a challenging issue. In this study, we began our study by using a murine contact hypersensitivity (CHS) model of ACD to examine the immunosuppressive effects of DYRK1B inhibition. We found that mice treated with a selective DYRK1B inhibitor show reduced ear inflammation. In addition, a significant reduction of Th1 and Th17 cells in the regional lymph node upon DYRK1B inhibition was observed by FACS analysis. Studies in vitro further revealed that DYRK1B inhibitor does not only suppressed Th1 and Th17 differentiation, but also promotes regulatory T cells (Treg) differentiation. Mechanistically, FOXO1 signaling was enhanced due to the suppression of FOXO1^Ser329^ phosphorylation in the presence of DYRK1B inhibitor. Therefore, these findings suggest that DYRK1B regulates CD4 T cell differentiation through FOXO1 phosphorylation and DYRK1B inhibitor has a potential as a novel agent for treatment of ACD.

## Introduction

Allergic contact dermatitis (ACD) is a form of inflammatory skin disease that is classified as a delayed or type IV hypersensitivity reaction and is mainly mediated by Th1/Th17 immune response^1–5^. ACD is clinically important because it can occur in general population and be common occupational skin disorders having a socioeconomic impact. Currently, topical corticosteroid is the first-line medical treatment for ACD, but systemic administration combining with immunosuppressive drugs such as azathioprine, cyclosporin or tacrolimus are used in widespread ACD, with greater than 20% of the body involvement^6,7^. However, increased risk of various adverse effects of these systemic drugs limits their use, and a rebound flare-up may occur upon drug cessation. Therefore, novel compounds that are effectively and safely used for treatment of ACD are of high clinical need.

DYRK1B belongs to the DYRK family within the CMGC (CDK, MAPK, GSK, and CLK) superfamily of protein kinases. DYRK1B derives its kinase function via co-translational autophosphorylation on a conserved tyrosine that resides within its catalytic domain. DYRK1B is highly expressed in skeletal muscle and the testes, and it has been previously reported that DYRK1B plays roles in the differentiation of these cells^8–10^. Moreover, it also has been reported that elevated DYRK1B expression promotes the survival of colon carcinoma, pancreatic ductal adenocarcinoma, non-small cell lung cancer, and rhabdomyosarcoma^11–14^. However, in contrast to the roles of DYRK1B in the systems described above, its role in the immune system remains elusive.

AZ-DYRK1B-33 is a small ATP-competitive inhibitor that potently inhibits the kinase activity of DYRK1B with an IC_50_ of 7 nM, with low cytotoxicity^15^. Oral administration of AZ-DYRK1B-33 at 10 mg/kg to BKS db/db mice for three weeks was well-tolerated^16^. In this study, we used AZ-DYRK1B-33 to evaluate the therapeutic potential of DYRK1B inhibition for ACD using a contact hypersensitivity (CHS) murine model and investigate its potential effects on the differentiation of CD4 T cells.

## Results

### Inhibition of DYRK1B attenuates inflammatory responses in murine CHS model and the number of Th1/Th17 cells in the regional lymph node

**Figure 1:**
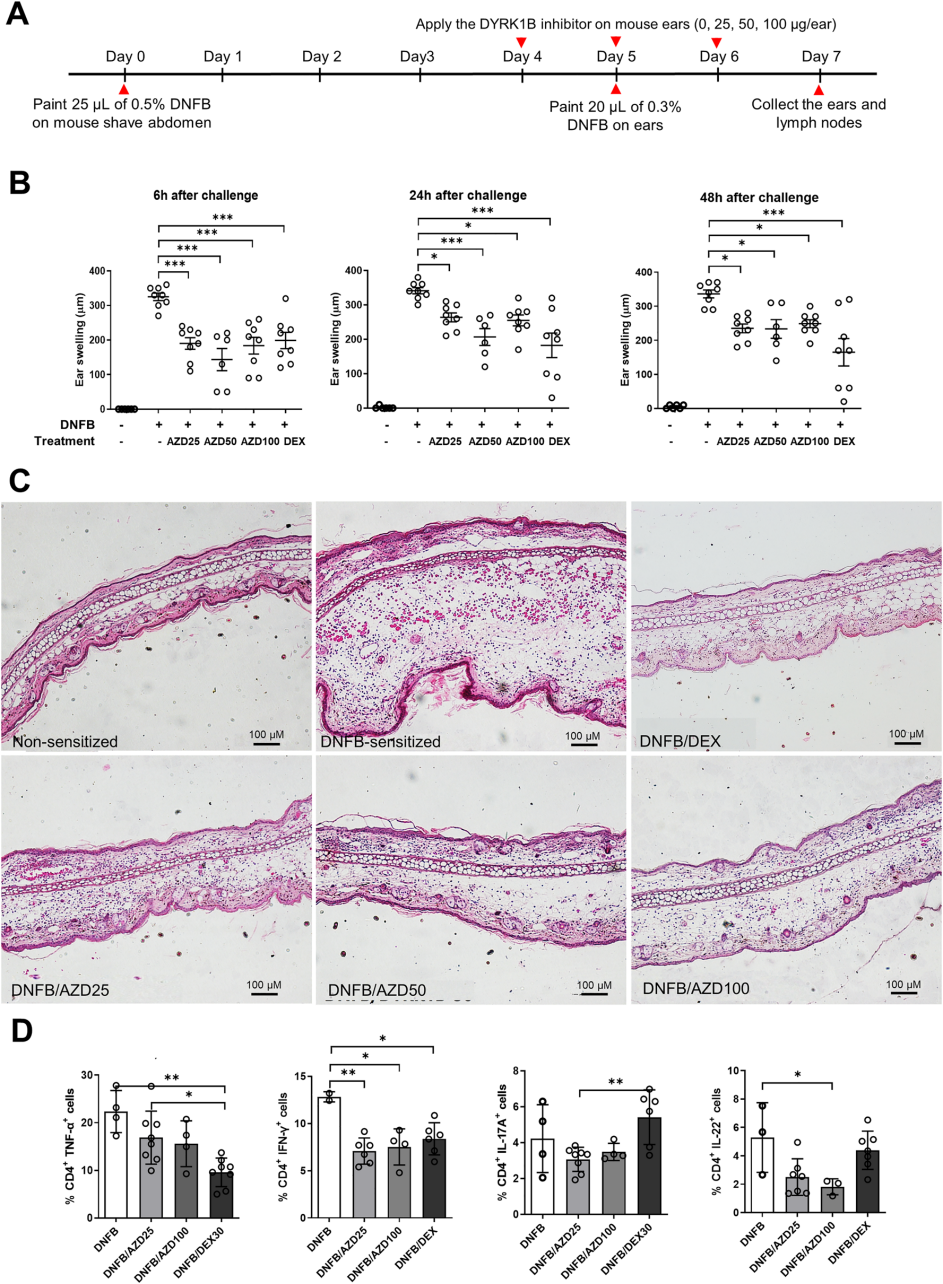
Inhibition of DYRK1B attenuates inflammatory responses in murine CHS model. (**A**) Schematic depicts the experiment design of a murine CHS model. (**B**) A selective DYRK1B inhibitor (AZD) at 25, 50, or 100 μg/ear or dexamethasone at 30 μg/ear was applied to both ears once daily on day 4–6. Mice that received vehicle alone were non-sensitized control. Ear thickness was measure at 6-, 24-, and 48-h post-challenge (n = 5). (**C**) Transverse sections of murine ears with no sensitization, DNFB sensitization, or DNFB sensitization together with topical application of 25, 50, or 100 μg/ear AZD or 30 μg/ear dexamethasone were compared at 48-h post-challenge. Tissues were stained with H&E. (**D**) Percentage of Th1 and Th17 in the regional lymph nodes of mice with no sensitization or DNFB sensitization together with topical application of 25 or 100 μg/ear AZD or 30 μg/ear dexamethasone. All graphs show mean ± SEM (*p < 0.05, **p < 0.01).

To evaluate the action of DYRK1B inhibitor toward ACD, the murine CHS model was sensitized with 2,4-dinitrofluorobenzene (DNFB)^17–20^ (Fig. 1A). We found that DNFB-treated mice developed marked ear swelling when compared to the non-sensitized control. Topical application of the DYRK1B inhibitor to the ears of the mice significantly reduced ear swelling within 6 h after DNFB challenge compared to the control mice (Fig. 1B). Notably, the decrease in the ear thickness of the DYRK1B inhibitor-treated mice were comparable to those treated with dexamethasone (Fig. 1B). Histologic examination of excised ear skin at 48 h after DNFB challenge revealed dermal thickening and inflammatory cells in DNFB-sensitized mice compared to non-sensitized mice. Ear edema was markedly decreased in both DYRK1B inhibitor-treated and dexamethasone-treated mice (Fig. 1C). Skin-infiltrating inflammatory cells were also decreased in the dermis of both DYRK1B inhibitor-treated and dexamethasone-treated mice compared to the DNFB-sensitized mice (Fig. 1C, Supplementary Fig. 1A,B).

According to the previous reports that CHS is mainly mediated by Th1/Th17 immune response, we further analyzed TNF-α, IFN-γ-, IL-17A, IL-22 producing CD4 T cells subpopulations in the regional lymph nodes from mice in each group by flow cytometry. We found that mice treated with the selective DYRK1B inhibitor show a significant decrease in Th1 (CD4^+^IFN-γ^+^ and CD4^+^TNF-α^+^) and Th17 (CD4^+^IL-17A^+^ and CD4^+^IL22^+^) population (Fig. 1D). These results together suggest that inhibition of DYRK1B suppresses skin-inflammation and Th1/Th17 immune response in murine CHS model.

### Inhibition of DYRK1B not only suppresses the differentiation of Th1 and Th17, but also enhances the differentiation of Treg in vitro

**Figure 2:**
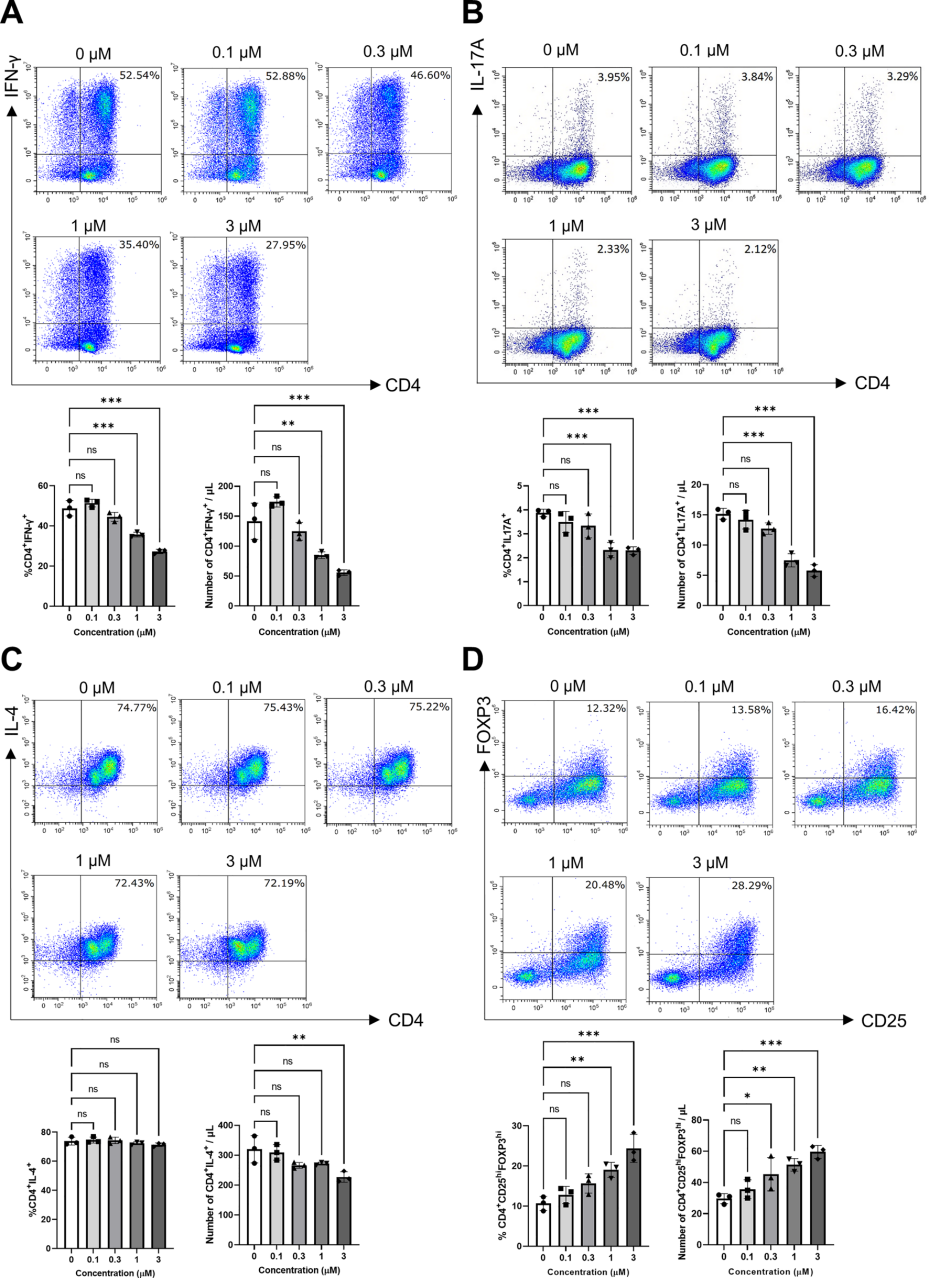
Inhibition of DYRK1B function suppresses Th1 and Th17, but enhances Treg differentiation. (**A–C**) Human naïve CD4^+^ T cells were stimulated by anti-CD3 and anti-CD28 and then differentiated under Th1-, Th17- and Th2-polarizing conditions, respectively, in the absence or presence of a selective DYRK1B inhibitor at 4 different concentrations for 96 h. The scatter dot plots represent data from triplicate sample and analyzed by flow cytometry for CD4^+^IFN-γ^+^, CD4^+^IL17A^+^, and CD4^+^IL4^+^, respectively. (**D**) Human naïve CD4^+^ T cells were stimulated by anti-CD3 and anti-CD28 and then differentiated in the presence of TGF-β1 and IL-2 with or without a selective DYRK1B inhibitor at 4 different concentrations for 96 h. The scatter dot plot represents data from triplicate samples as analyzed by flow cytometry for iTreg (CD4^+^CD25^+^FOXP3^+^) population. The results are summarized in the bar graphs, and data are presented as mean ± SEM (*p < 0.05, **p < 0.01, ***p < 0.001), *ns* non-significant).

Given that mice treated with DYRK1B inhibitor affects the Th1/Th17 CD4 T cell subpopulation in CHS model, we next questioned the role of DYRK1B in CD4 T cells differentiation. To this end, we isolated the naïve CD4^+^ T cells from human PBMCs and cultured under Th1-, Th2-, Th17-, and Treg-polarizing conditions in the absence or presence of a selective DYRK1B inhibitor. We found that the addition of the DYRK1B inhibitor significantly suppressed the percentage of Th1 (CD4^+^IFN-γ^+^) and Th17 (CD4^+^IL17A^+^) differentiation (Fig. 2A,B), but not that of Th2 (CD4^+^IL-4^+^) (Fig. 2C). Interestingly, we also found that inhibition of DYRK1B significantly enhanced in vitro induced Treg (iTreg, CD4^+^CD25^hi^FOXP3^hi^) differentiation in a concentration-dependent manner (Fig. 2D). We further conducted qRT-PCR analysis of naïve CD4^+^ T cells cultured under Treg-polarized condition and treated with DYRK1B inhibitor at different time points. We found that *FOXP3* expression was upregulated approximately three times at 24 h with a slight subsequent decrease that remained constant for at least 96 h (Supplementary Fig. 2A). Moreover, cell viability was not affected in the presence of the DYRK1B inhibitor (Supplementary Fig. 3A,B). Taken together, these results demonstrate that inhibition of DYRK1B not only suppressed Th1 and Th17 differentiation, but also enhanced Treg differentiation in vitro.

### DYRK1B regulates CD4 T cell differentiation through the suppression of FOXO1 activity

**Figure 3:**
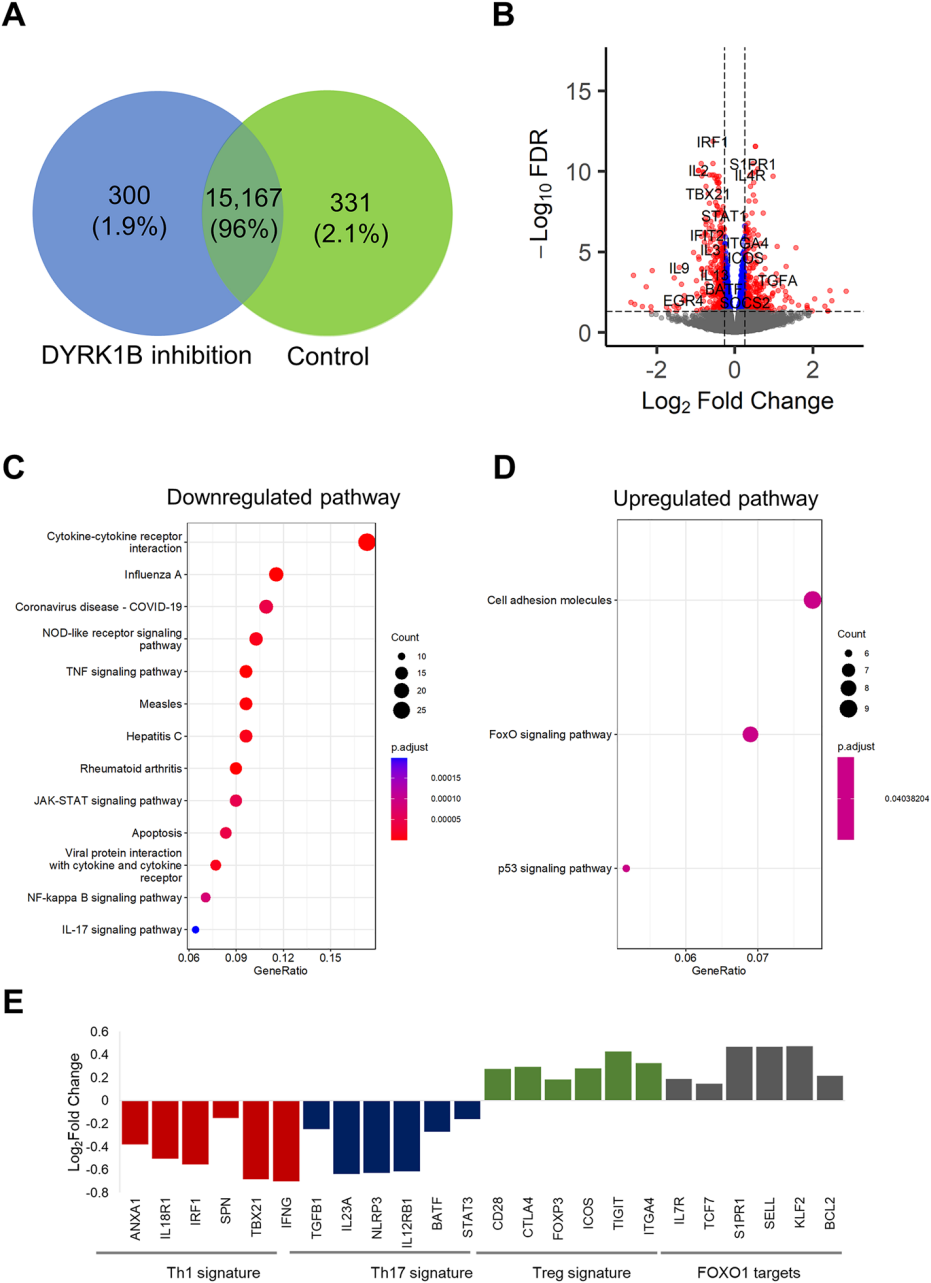
DYRK1B inhibition regulates naïve CD4^+^ T cell differentiation by enhancing FOXO1 activity, which results in the suppression of Th1 and Th17 differentiation signaling. (**A**) Venn diagram showing the number of genes that are individually expressed in the DYRK1B inhibition (AZ-DYRK1B-33, 1 µM) and control subsets. (**B**) A volcano plot shows differentially expressed stimulated naïve CD4^+^ T cells compared between the absence and presence of a selective DYRK1B inhibitor. The data point above the significance threshold (FDR < 0.05) are marked in blue (− 1.2 < Log2 fold change < 1.2) and red (− 1.2 > Log2 fold change > 1.2). (**C,D**) A dot plot shows KEGG enrichment analysis of upregulated and downregulated pathways found after addition of a selective DYRK1B inhibitor compared to control, respectively (FDR < 0.05). The size of a point reflects the number of annotated genes. (**E**) A histogram shows significantly upregulated and downregulated Th1, Th17, Treg signature genes, and FOXO1 target genes after the addition of a selective DYRK1B inhibitor compared to control (FDR < 0.05).

To further elucidate the underlying molecular mechanisms by which DYRK1B inhibition suppresses Th1 and Th17 differentiation and promotes Treg differentiation in vitro, we performed RNA sequencing of stimulated naïve CD4^+^ T cells in the presence of IL2 and TGF-β and examined the effect of the addition of DYRK1B inhibitor at the final concentration of 1 μM. We obtained read count ~ 43–60 million per sample. Among these reads, ~ 41–57 million reads per sample were successfully mapped to the reference human genome. We then generated expression matrix and conducted downstream differential expression gene (DEG) analysis. We found 300 DEGs specific to samples treated with DYRK1B inhibitor and 331 DEGs specific control samples respectively (Fig. 3A). Volcano plot of DEGs further revealed upregulation of Treg signature genes, such as *CTLA4* and *ICOS*, but downregulation of several Th1 and Th17 signature genes, such as *TBX21*, *IFNG*, and *IL17A* (Fig. 3B). Moreover, Gene Sets Enrichment Analysis (GSEA) using the KEGG terms showed significant downregulation of the TNF and Th17 signaling pathways (Fig. 3C), and significant upregulation of the FOXO signaling pathway (Fig. 3D). It should be noted that the central transcription factor FOXO1 of FOXO signaling pathway was previously reported inhibiting Th1 and Th17 differentiation via the suppression of *TBX21* and RORγT, while promoting Treg differentiation via *FOXP3* induction^21–24^. We further plotted the change of expression level of representative Th1 signature genes (*ANXA1*, *IL18R1*, *IRF1*, *SPN*, *TBX21*, *IFNG*), Th17 signature genes (*TGFB1*, *IL23*, *NRLP3*, *IL12RB1*, *BATF*, *STAT3*), Treg signature genes (*CD28*, *CTLA4*, *FOXP3*, *ICOS*, *TIGIT*, *ITGA4*) and FOXO1 target genes (*IL7R*, *TCF7*, *S1PR1*, *SELL*, *KLF2*, and *BCL2*) and found that in consistent with the above findings, DYRK1B inhibition suppressed the expression of Th1 and Th17 genes, while enhanced Treg and FOXO1 target genes (Fig. 3E). These results together suggest that DYRK1B regulates naïve CD4^+^ T cells differentiation into effector Th1, Th17, and Treg via the control of FOXO1.

### Inhibition of DYRK1B decreases phosphorylation of FOXO1 at Ser329 and promotes FOXO1 transcriptional activity

**Figure 4:**
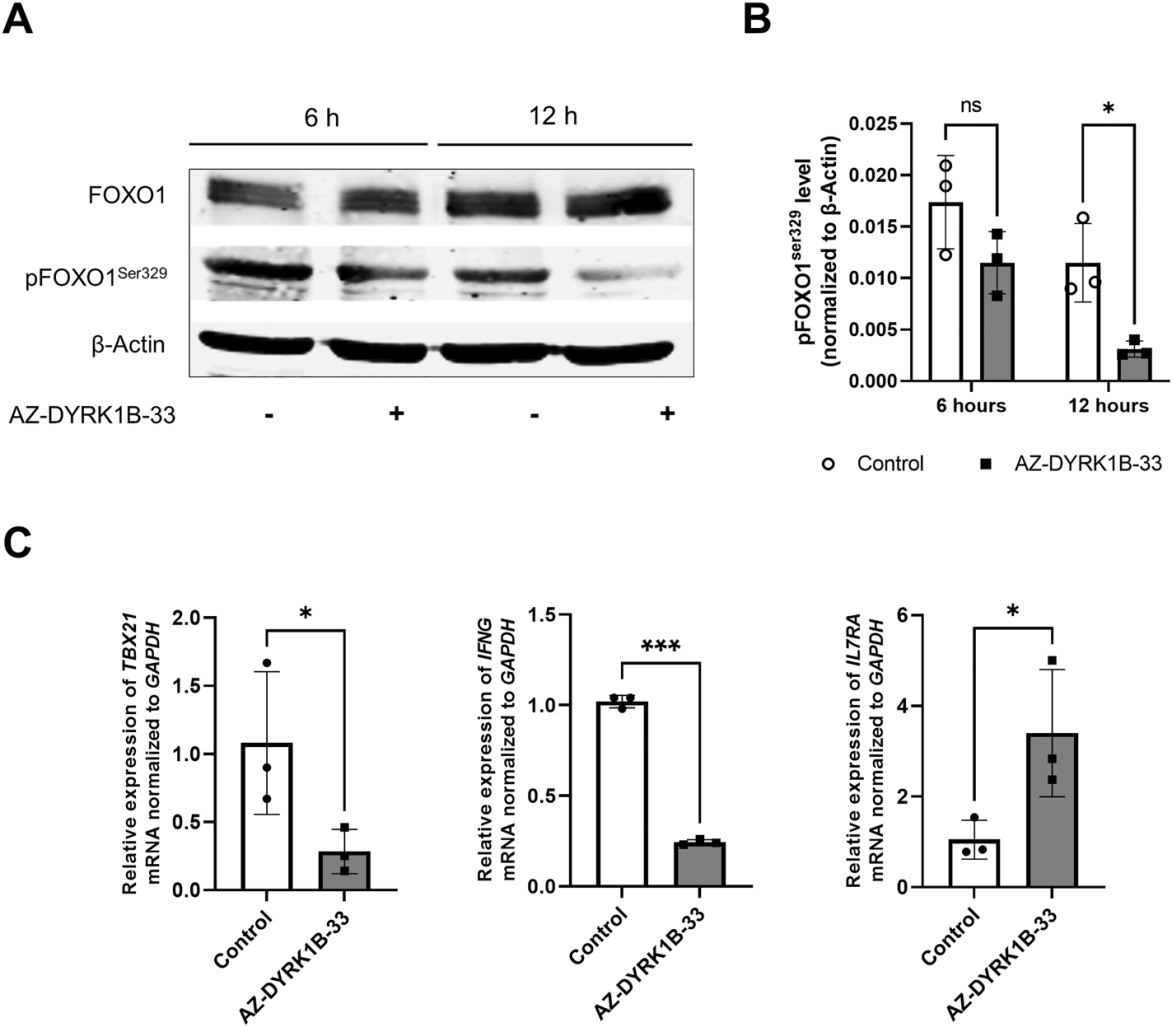
DYRK1B inhibition reduces phosphorylation of FOXO1 and enhances *FOXP3* expression. (**A**) Human naïve CD4^+^ T cells stimulated under Treg-polarizing conditions in the absence or presence of a selective DYRK1B inhibitor (AZ-DYRK1B-33, 1 µM) for 6 or 12 h. The extracted proteins were analyzed by immunoblotting with anti-FOXO1 or anti-pFOXO1^Ser329^. (**B**) pFOXO1^Ser329^ levels from (**A**) were quantified using Image Studio version 5.2 software and are summarized in a bar graph. (**C**) Relative mRNA expression levels of FOXO1 target genes in naïve CD4^+^ T cells stimulated under Treg-polarizing conditions in the absence or presence of a selective DYRK1B inhibitor (AZ-DYRK1B-33, 1 µM) for 24 h were analyzed by qRT-PCR. The results are summarized in the bar graphs, and the data are presented as mean ± SEM (*p < 0.05, ***p < 0.001).

Given that our transcriptomic analysis results suggested activation of the FOXO signaling pathway upon DYRK1B inhibition, and it has been previously reported that FOXO1 could be phosphorylated by DYRK1 family at Ser329 that inhibits its transcriptional activity and nuclear exlusion^25–27^, we next examined FOXO1^Ser329^ phosphorylation in stimulated naïve CD4^+^ T cells in the presence of IL2 and TGF-β with or without DYRK1B inhibitor by Western blotting. We found that p-FOXO1^Ser329^ level was significantly reduced upon addition of the DYRK1B inhibitor (Fig. 4A,B), suggesting FOXO1 activation. In this light, we noted that it has been previously reported that FOXO1 directly suppresses *TBX21* and *IFNG* expression, whereas promotes *IL7RA* expression^13,28,29^ and therefore subsequently conducted quantitative RT-PCR analysis on these genes. Consistently, we found that *TBX21* and *IFNG* expression were suppressed and *IL7R*A expression was enhanced upon DYRK1B inhibition (Fig. 4C). These results together suggest that DYRK1B regulates CD4 T cell differentiation through the phosphorylation of FOXO1 at inhibitory Ser329.

## Discussion

CHS is an animal model for human ACD, in which its pathophysiology is mainly mediated by CD4 T cells. It has long been known about the major involvement of Th1 immune response in CHS. Moreover, recent evidences suggested that Th17 also plays an important role too. For example, it has been shown that the presence of Th17 in a skin lesion increases allergic inflammation^4,5^. In this study, we have investigated the therapeutic effect of the DYRK1B inhibitor in CHS mice. We showed that DYRK1B inhibition not only attenuates inflammation in the skin, but also suppresses Th1/Th17 response in regional lymph node in CHS mice. We therefore speculate that the immunosuppressive effect of DYRK1B inhibitor is potentially mediated through the control of Th1/Th17 differentiation in CHS model. However, it should be noted that we could not rule out the possibility that DYRK1B inhibitor may also affect cells other than T cells that contribute to the pathophysiology of CHS, such as keratinocytes, fibroblasts and other immune cells. This issue could be confirmed by the using of DYRK1B conditional knockout mice in the future.

After their activation by cognate antigens, naïve CD4^+^ T cells can differentiate into either specific helper T cells (Th), including Th1, Th2, and Th17, or Treg depending on the cytokine milieu program. Th cells serve as a key modulator in the activation of macrophages, cytotoxic T cells, and B cell maturation and function. In contrast, Treg plays a suppressive role in the maintenance of peripheral immune tolerance^30,31^. Consequently, an imbalance between Th1, Th2, or Th17 and Treg associates with various allergic inflammation and autoimmune diseases^32–39^. Interestingly, using in vitro T cell differentiation system, we found that inhibition of DYRK1B did not only suppress Th1 and Th17 differentiation, but also enhanced Treg differentiation. We therefore speculate that DYRK1B inhibitor may also have a potential to restore self-tolerance in immune dysregulation disorders that are mainly mediated through the impairment of Treg.

In breast and ovarian cancer cells, the phosphorylation of FOXO1 by DYRK1B resulted in decreasing transcriptional activity^26,27^. Unlike cancer cells, there is no evidence that suggests FOXO1 is downstream from DYRK1B in the immune system. This raises the question whether DYRK1B is an upstream kinase of FOXO1 in CD4 T cells. Based on our biochemical analysis, DYRK1B phosphorylates FOXO1 at Ser329 that subsequently inhibits FOXO1 transcriptional activity in CD4 T cells. FOXO1 is a transcription factor that plays an essential role in a variety of cellular processes, including metabolism, cell cycle progression, differentiation, and apoptosis^40–43^. T cell-specific deletion of *Foxo1* led to autoimmunity because of decreasing FOXP3^+^ Treg cells while increasing T follicular helper in vivo^44–46^. The upregulation of FOXO1 could lessen inflammation in liver ischemia and reperfusion injury through the downregulation of NF-κB and NLRP3 inflammasome^47^. In CD4^+^ T cells, FOXO1 directly bound the promoter region of *Ifng* and suppressed its gene expression^48^. Furthermore, in the presence of TGF-β, T cell-specific deletion of *Foxo1* results in impairment of Treg differentiation and misdirection toward Th1^44^. In Th17 cells, FOXO1 bound the DNA binding domain of RORγt that led to the suppression of RORγt activity and hence decreasing Th17 differentiation^22^. In contrast, genetic deletion of *Foxo1* in CD4^+^ T cells increased IL17A expression and Th17 differentiation^49^. Moreover, *Foxo1* was directly repressed by miR-183C driving Th17 cell pathogenic function^50^. In Tregs, FOXO1 bound the promoter of *Foxp3* and induced its gene expression, thus facilitated Treg cell differentiation^23,24^. Taken these reports together with our in vitro data suggested that the inhibition of DYRK1B promoted Treg differentiation while suppressed Th1 and Th17 differentiation through the regulation of FOXO1 inhibitory phosphorylation in vitro. However, the effect of DYRK1B on the phosphorylation of FOXO1 in vivo has not been confirmed and warrants further investigation.

DYRK1B was previously reported to be a key regulator of skeletal muscle cell differentiation^51^. Moreover, it also plays important roles in cell-cycle progression and the survival of several cancer cell lines^11–14^. To activate its kinase function, DYRK1B auto-phosphorylates its tyrosine residue (Y273) in the activation loop during translation^8–10^. Furthermore, in human embryonic kidney (HEK 293) cells, a recent study suggests that DYRK1B is also activated via the phosphorylation of serine residue (S421) by ERK2^52^. Notably, it has been reported that activation of the MAP/ERK2 pathway activates DYRK1B kinase function in cancer cells and further phosphorylates its downstream proteins, such as FOXO1^26,27,52^. Interestingly, it was previously reported that activation of ERK2 is required for Th1 differentiation^53^. Moreover, it was also reported that inhibition of ERK2 suppressed Th17 differentiation, but induced FOXP3 expression and Treg differentiation^54^. Given that our results suggest similar suppression of effector Th1 and Th17 differentiation and enhancement of Treg differentiation upon DYRK1B inhibition, we speculate that ERK2 might be the upstream signaling molecule of DYRK1B in CD4 T cell differentiation. Further investigation should be pursued to confirm in the future.

Given that our data (Supplementary Fig. 3A,B) and a previous report^15^, both indicate that inhibition of DYRK1B function by AZ-DYRK1B-33 had no clear cytotoxicity in vitro even at high concentration and the topical application of AZ-DYRK1B-33 in mice in vivo did not show any obvious side effects, we believe that this compound is a promising candidate drug for ACD. However, it should be noted that DYRK1B is highly expressed in skeletal muscle and testes. In skeleton muscle, DYRK1B was activated by Rho-family protein to promote myoblast differentiation^51^. In testes, DYRK1B interacted with Cold-inducible RNA-binding protein (Cirp) modulating p27 and cyclin D1 stability to fine-tune the proliferation of undifferentiated spermatogonia^55^. We therefore could not exclude the possibility that myopathy and impairment of spermatogenesis might occur upon long-term treatment. In addition, previous studies also suggested that DYRK1B may play roles in adipogenesis and glucose metabolism. It is therefore also possible that metabolic syndrome, such as central obesity, hypertension, and/or hyperglycemia, could develop upon the administration of DYRK1B inhibitor^56,57^. Further studies on the pharmacokinetics and long-term in vivo toxicity are warranted to unravel the therapeutic potential of DYRK1B inhibitor in human ACD.

## Materials and methods

### Ethics declarations

All the animal procedures were performed in accordance with the relevant guidelines and regulations, and were approved by the Siriraj Animal Care and Use Committee of the Faculty of Medicine Siriraj Hospital, Mahidol University, Bangkok, Thailand (assurance number COA no. 002/2565). All the human experimental protocols and procedures were performed in accordance with the relevant guidelines and regulations, and were approved by the Human Research Protection Unit of the Faculty of Medicine Siriraj Hospital, Mahidol University, Bangkok, Thailand (assurance number COA no. 663/2564[IRB1]).

### Mice

Female 8-week-old C57BL/6NJc1 mice were purchased from Nomura Siam International (Bangkok, Thailand). All mice were maintained in a specific pathogen-free condition with free access to standard rodent feed and water. All procedures were conducted in accordance with the guidelines of Mahidol University and the National Research Council of Thailand, and in complied with the ARRIVE guidelines. The number of animals used in each experiment was determined according to the number of animals used in similar experiments conducted in previously published studies. All experimental protocols were approved by the Siriraj Animal Care and Use Committee of the Faculty of Medicine Siriraj Hospital, Mahidol University, Bangkok, Thailand (assurance number COA no. 002/2565).

### Skin contact hypersensitivity model

DNFB was used as a hapten for inducing murine CHS as described by Honda et al. and Thitilertdecha et al.^19,20^. All mice were sensitized by painting 25 μL of 0.5% DNFB diluted in acetone and olive oil (4:1, v/v) on the shaved abdomen (day 0) with an average coverage area of 2 cm^2^. On day 5, mice were elicited by painting 20 μL of 0.3% DNFB in the same vehicle on the dorsal and ventral pinna of both ears. For topical application of chemical and drug compounds, mouse pinnae were painted with either AZ-DYRK1B-33 (25, 50, or 100 μg/ear, treatment groups, HY-117391, MedChemExpress), dexamethasone (30 μg/ear, positive control), or no treatment (vehicle alone, sensitized control) starting on day 4 for 3 consecutive days. Mice sensitized and elicited with vehicle alone were used as a non-sensitized control. The ear thickness of each ear of each individual mouse was measured at pre-challenge and at 6-, 24-, and 48-h post-challenge using a Vernier caliper (Mitutoyo, Kanagawa, Japan).

### Histopathologic examination

At 48-h post-challenge, all mice were sacrificed by intraperitoneal injection of thiopental sodium (50 mg/kg). The mouse ears were resected, fixed with 4% paraformaldehyde, embedded in paraffin. Sections (5 μm thick) were stained with hematoxylin and eosin (H & E) for histopathologic evaluation of CHS. Inflammatory cell infiltration in each section was quantified by counting in each visual field and divided by area of quantification using Image J.

### Human blood

Human blood samples were obtained from healthy donors after obtaining written informed consent, and the procedures were conducted in accordance with the requirements of the Human Research Protection Unit of the Faculty of Medicine Siriraj Hospital. All experimental protocols were approved by the Human Research Protection Unit of the Faculty of Medicine Siriraj Hospital, Mahidol University, Bangkok, Thailand (assurance number COA no. 663/2564[IRB1]).

### Cell isolation and in vitro differentiation

Human PBMCs were isolated from healthy donor blood using Ficoll-Hypaque (IsoPrep; Robbins Scientific Corporation, San Diego, CA, USA) gradient centrifugation. Naïve CD4^+^ T cells were isolated via magnetic cell sorting using a human naïve CD4^+^ T cell isolation kit (MACs; Miltenyi Biotec, Bergisch Gladbach, Germany). Complete RPMI supplemented with 10% FBS, 50 µM 2-mercaptoethanol, 100 U/mL penicillin, 100 µg/mL streptomycin, and 10 mM HEPES was used as a culture medium. The isolated naïve CD4^+^ T cells were stimulated with immobilized anti-CD3 Ab (plates coated with 10 µg/mL in PBS, 16-0037-85 eBioscience) and 1 µg/mL soluble anti-CD28 (16-0289-85, eBioscience) for 96 h before analysis. The culture medium was supplemented with 15 ng/mL TGF-β1 (580704, BioLegend) and 100 U/mL IL-2 (589104, BioLegend) for Treg differentiation; with 10 ng/mL IL-12 (PHC1124, Gibco), 5 µg/mL anti-IL-4 Ab (16–7048-85, eBioscience), and 50 U/mL IL-2 (589104, BioLegend) for Th1 differentiation; with 30 ng/mL IL-4 (PHC0044, Gibco), 5 µg/mL anti-IFN-γ Ab (16-7318-81, eBioscience), and 50 U/mL IL-2 (589,104, BioLegend) for Th2 differentiation, and with 5 ng/mL TGF-β1 (580704, BioLegend), 25 ng/mL IL-6 (PHC0066, Gibco), 50 ng/mL IL-23 (PHC9324, Gibco), 5 µg/mL anti-IL-4 Ab (16-7048-85, eBioscience), and 5 µg/mL anti-IFN-γ Ab (16–7318-81, eBioscience) for Th17 differentiation. In each different culture condition, the DYRK1B inhibitor (HY-117391, MedChemExpress) was added on day 0 at the concentration of 0.1, 0.3, 1, or 3 µM.

### Cell staining and flow cytometry

Surface staining (CD4-FITC; 555346 BD Bioscience and CD25-PE; 555432 BD Bioscience) was performed in PBS supplemented with 2% FBS for 30 min at ambient temperature. For intracellular staining of iTreg, the cells were fixed and permeabilized using a BD Pharmingen Stain Buffer Set (BD Biosciences, Franklin Lakes, NJ, USA), followed by washing and staining with APC-conjugated anti-human FOXP3 (560045, BD Bioscience) for 30 min. For evaluation of Th1, Th2, and Th17, the cells were stimulated with 50 ng/mL PMA (P1585, Sigma-Aldrich) and 1 μg/mL ionomycin (I3909, Sigma-Aldrich) for 4 h at 37 °C in the presence of GolgiPlug protein transport inhibitor (502301KZ, BD Biosciences) before surface staining. BD Cytofix/Cytoperm (BD 554714, BD Biosciences) was used as a fixing and permeabilizing reagent for intracellular cytokine staining. The cells were then washed and stained with APC-conjugated anti-human IFN-γ (17-7319-82, eBioscience), IL-4 (17-7049-81, eBioscience), or IL-17A (17-719-42, eBioscience) and incubated on ice for 30 min. Flow cytometry was performed using a Cytoflex flow cytometer (Beckman Coulter Life Sciences, Indianapolis, IN, USA), and the data were analyzed using CytExpert software (Beckman Coulter Life Sciences).

### RNA extraction, cDNA synthesis, and quantitative RT-PCR

The cells were harvested and lysed in Trizol reagent (Invitrogen, Carlsbad, CA, USA). RNA was extracted using an RNAeasy Mini Kit (Qiagen, Hilden Germany), and then assessed for concentration and purity using a Nanodrop spectrophotometer (Thermo Fisher Scientific, Waltham, MA, USA). cDNA synthesis was performed using iScript Reverse Transcription Supermix (Bio-Rad Laboratories, Hercules, CA, USA). KAPA SYBR FAST qPCR Master Mix (Sigma-Aldrich) was used for quantitative PCR, which was run on QuantStudio 5 software (Thermo Fisher Scientific) and analyzed using QuantStudio design and analysis software version 1.3.1 (Thermo Fisher Scientific). Gene expression levels were normalized to *GAPDH*. Primer used for PCR amplification are forward: 5ʹ TTCATCTGTGGCATCATCCG 3ʹ, reverse: 5ʹ TCGCATGTTGTGGAACTTGA 3ʹ for *FOXP3*, forward: 5ʹ TGTGGAGACCATCAAGGAAGACA, reverse: 5ʹ GGCGACAGTTCAGCCATCAC 3ʹ for *IFNG*, forward: 5ʹ AAAGCTCCAACCGGCAGCAA 3ʹ, reverse: 5ʹ CAAGATGACCAACAGAGCGAC 3ʹ for *IL7RA*, forward: 5ʹ GCGTGTCCCCCTATCCTTCC 3ʹ, reverse: 5ʹ GGGGGCCTTCTCAGTCCTTC 3ʹ for *TBX21* and forward: 5ʹ AAATTCCATGGCACCGTCAAG 3ʹ, reverse: 5ʹ TGGTTCACACCCATGACG-AA 3ʹ for *GAPDH*.

### RNA sequencing and analysis

Human naïve CD4^+^ T cells were stimulated with anti-CD3 and anti-CD28 under Treg-polarizing conditions for 24 h in the absence or presence of 1 µM DYRK1B inhibitor. Total RNA were isolated using an RNAeasy Mini Kit (Qiagen), and the RNA concentration was assessed using a Qubit™ RNA Broad Range Assay Kit (Thermo Fisher Scientific). Bioanalyzer analysis using an Agilent 2100 analyzer (Agilent Technologies, Santa Clara, CA, USA) showed the RNA integrity (RIN) values for all samples to be ≥ 7.8. mRNA were enriched at the poly-A tail using oligo-dT attached beads. Libraries were prepared using an Ultra II Directional RNA Library Prep Kit for Illumina. Paired-end libraries were sequenced using an Illumina HiSeq™ system. The original raw data were transformed to sequenced reads via base-calling. Raw data were recorded in a FASTQ file that contained sequence information (reads) and corresponding sequencing quality information. Index of the reference genome was built using Hisat2 v2.0.5 and paired-end clean reads were aligned to the reference human genome (hg38) using Hisat2 v2.0.5. Differential gene expression analysis was performed using edgeR software (v3.38.2). The resulting p-values were adjusted using the Benjamini–Hochberg method for controlling the FDR. Genes with an FDR < 0.05, and a Log2 fold change of <  − 1.2 or > 1.2 were defined as differentially expressed. The GO database, the KEGG database, and the Reactome Pathway database were used for gene set enrichment analysis using clusterProfiler R software. The model used, the company that is was purchased from, and the headquarters location of that company need to be sourced and entered for every product in the preceding paragraph.

### Western blot analysis

Cells were harvested, washed twice with cold PBS, lysed in RIPA lysis buffer supplemented with protease and phosphatase inhibitor cocktail (Thermo Fisher Scientific), and incubated on ice for 30 min with vortexing for 10 s at 15-min intervals. The protein concentration was determined using a Bradford protein assay (Bio-Rad Laboratories), after which equal amounts of total protein were run on poly-acrylamide gels that were prepared in-house. After electrophoresis, the proteins were transferred using an iBlot semi-dry transfer system (Thermo Fisher Scientific). The following primary antibodies was used: mouse anti-human FOXO1 (1452T, Cell Signaling), rabbit anti-human pFOXO1^Ser-329^ (PA5-38275, Invitrogen) and mouse anti-human β-actin (sc-47778, Santa Cruz Biotechnology, Inc.). The following secondary antibodies were used: goat anti-rabbit IgG H&L (IRDye® 800CW, ab216773, Abcam) and goat anti-mouse IgG H&L (IRDye® 680RD, ab216776, Abcam). The IRDye® infrared fluorescent dye signal on the cellulose membranes was detected and calculated using an Odyssey® CLX imaging system and Image Studio software, respectively.

### Statistical analysis

All data summarized in the bar/line graphs are presented as mean ± SEM. Statistical comparisons were performed using Student’s unpaired t-test (2-tailed) or 1- or 2-way ANOVA with subsequent Tukey’s test. These comparisons were performed using GraphPad Prism (GraphPad Software, San Diego, CA, USA). A p-value < 0.05 was considered statistically significant.

## Supplementary Information

Supplementary Legends.

Supplementary Figures.

## Data Availability

The RNA-seq datasets generated and analyzed during the current study are available in the Gene Expression Omnibus (GEO) repository under the accession codes: GSE215457, https://www.ncbi.nlm.nih.gov/geo/query/acc.cgi?acc=GSE215457.
