# Supplementary material for: Inhibition of DYRK1B suppresses inflammation in allergic contact dermatitis model and Th1/Th17 immune response
Source: Sci Rep. 2023 Apr 29;13:7058. doi: 10.1038/s41598-023-34211-x (PMC10148813; doi:10.1038/s41598-023-34211-x)
Supplement: Supplementary file 1 — Supplementary Legends. [file 41598_2023_34211_MOESM1_ESM.docx]

**Supplementary Figure 1. Inflammatory cells in the dermis of the excised ear. A,** Transverse sections of murine ears with no sensitization, DNFB sensitization, or DNFB sensitization together with topical application of 25, 50, or 100 μg/ear AZ-DYRK133 (AZD) or 30 μg/ear dexamethasone (DEX) of the same tissue slides as shown in figure 1C were taken with a 40× objective lens. **B,** Inflammatory cell infiltration in each section was quantified by counting in each visual field and divided by area of quantification using Image J. The results are summarized in the bar graph, and the data are presented as mean±SEM (**p* < 0.05, ***p* < 0.01, ****p* < 0.001).

**Supplementary Figure 2. *FOXP3* in human naïve CD4^+^** **T cell. A,** Relative mRNA expression levels of *FOXP3* in human naïve CD4^+^ T cell stimulated under Treg-polarizing conditions in the absence or presence of a selective DYRK1B inhibitor (AZ-DYRK1B-33, 1 µM) for 24 to 96 h were analyzed by qRT-PCR.

**Supplementary Figure 3. Cell viability analysis. A and B,**Human naïve CD4^+^ T cells were stimulated with anti-CD3 and anti-CD28 and differentiated under Treg-polarizing conditions in the absence or presence of a selective DYRK1B inhibitor (AZ-DYRK1B-33) at 0.01, 0.1, 1, 5 and 10 µM for 96 h. Propidium iodide (PI) was used for cell viability staining. Histograms represent data from triplicated samples analyzed by flow cytometry and are summarized as bar graph. Data represent mean ±SD *p < 0.05, **p < 0.01, ***p < 0.001.

**Supplementary Figure 4.** A and B, A representative whole blot for western blot analysis of each protein in Figure 4A. Cells were stimulated with anti-CD3 and anti-CD28 and differentiated under Treg-polarizing conditions in the absence or presence of a selective DYRK1B inhibitor (AZ-DYRK1B-33) at 1 µM for 6 and 12 h. The extracted proteins were analyzed by immunoblotting with anti-FOXO1 or anti-pFOXO1^Ser329^.  pFOXO1^Ser329^levels were quantified using Image Studio version 5.2 software
