# Supplementary material for: Inhibition of DYRK1B suppresses inflammation in allergic contact dermatitis model and Th1/Th17 immune response
Source: Sci Rep. 2023 Apr 29;13:7058. doi: 10.1038/s41598-023-34211-x (PMC10148813; doi:10.1038/s41598-023-34211-x)
Supplement: Supplementary file 2 — Supplementary Figures. [file 41598_2023_34211_MOESM2_ESM.pdf]

Supplementary Information

Inhibition of DYRK1B suppresses inflammation in allergic contact dermatitis model and Th1/Th17 immune response

Thamrong Wongchang[1,2], Panwadee Pluangnooch[1], Suradej Hongeng[3,4], Adisak Wongkajornsilp[1], Dean Thumkeo[5], Kitipong Soontrapa[1]

[1] Department of Pharmacology, Faculty of Medicine Siriraj Hospital, Mahidol University, Bangkok, Thailand
[2] Division of Pharmacology, Department of Pharmaceutical Care, School of Pharmaceutical Sciences, University of Phayao, Phayao, Thailand
[3] Department of Pediatrics, Faculty of Medicine Ramathibodi Hospital, Mahidol University, Bangkok, Thailand
[4] Excellent Center for Drug Discovery, Mahidol University, Bangkok, Thailand
[5] Department of Drug Discovery Medicine, Medical Innovation Center, Kyoto University Graduate School of Medicine, Kyoto, Japan

Correspondence to:
Kitipong Soontrapa, MD, PhD
Assistant Professor of Pharmacology
Department of Pharmacology
Faculty of Medicine Siriraj Hospital, Mahidol University
2 Wanglang Road, Bangkoknoi, Bangkok 10700, Thailand
Tel: (+66) 2-419-7565; Mobile: (+66) 81-934-1734; Fax: (+66) 2-411-5026
E-mail: kitipong.soo@mahidol.ac.th

Supplementary Figure 1

A

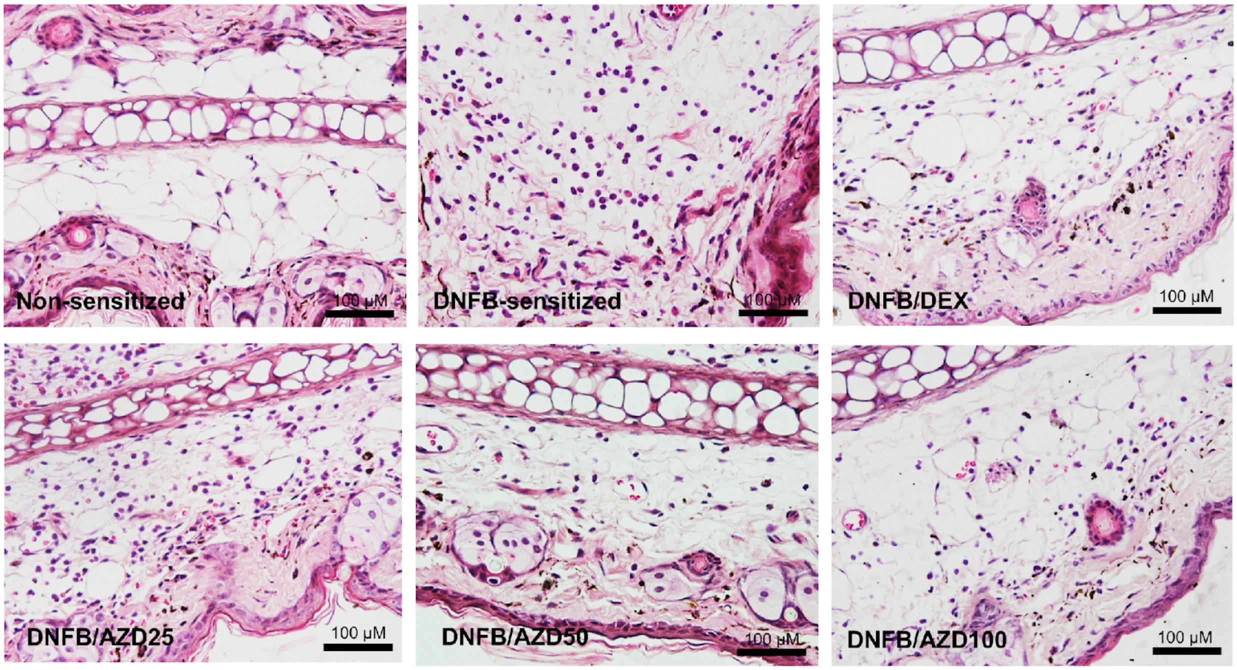

B

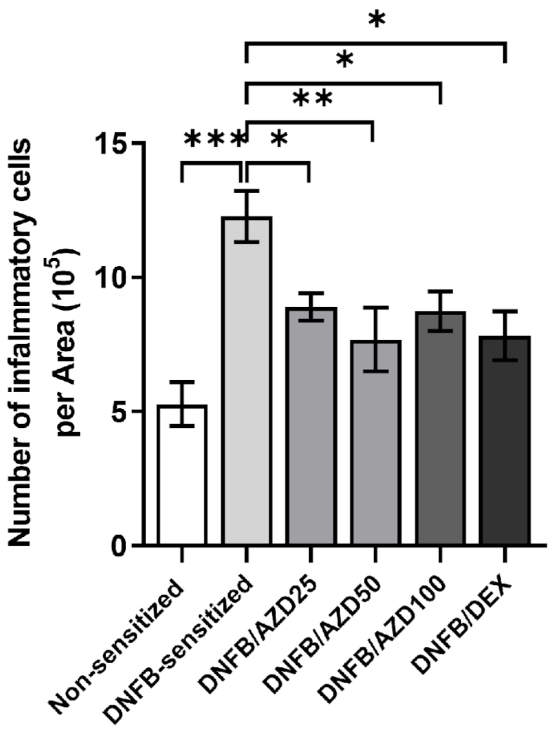

Supplementary Figure 1. Inflammatory cells in the dermis of excised ear skin. A, Transverse sections of murine ears with no sensitization, DNFB sensitization, or DNFB sensitization together with topical application of 25, 50, or 100 μg/ear AZ-DYRK133 (AZD) or 30 μg/ear dexamethasone (DEX) of the same tissue slides as shown in figure 1C were taken with a 40x objective lens. B, Inflammatory cell infiltration in each section was quantified by counting in each visual field and divided by area of quantification using Image J. The results are summarized in the bar graph, and the data are presented as mean±SEM (*$p < 0.05$, **$p < 0.01$, ***$p < 0.001$).

Supplementary Figure 2

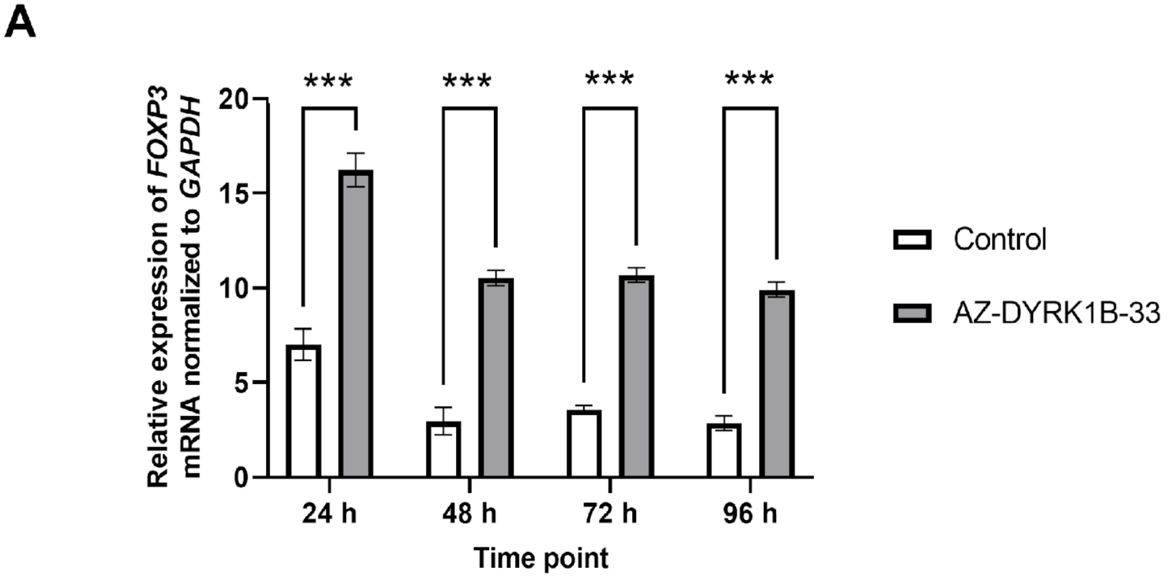

Supplementary Figure 2. *FOXP3* in human naïve CD4$^+$ T cell. A, Relative mRNA expression levels of *FOXP3* in human naïve CD4$^+$ T cell stimulated under Treg-polarizing conditions in the absence or presence of a selective DYRK1B inhibitor (AZ-DYRK1B-33, 1 μM) for 24 to 96 h were analyzed by qRT-PCR.

Supplementary Figure 3

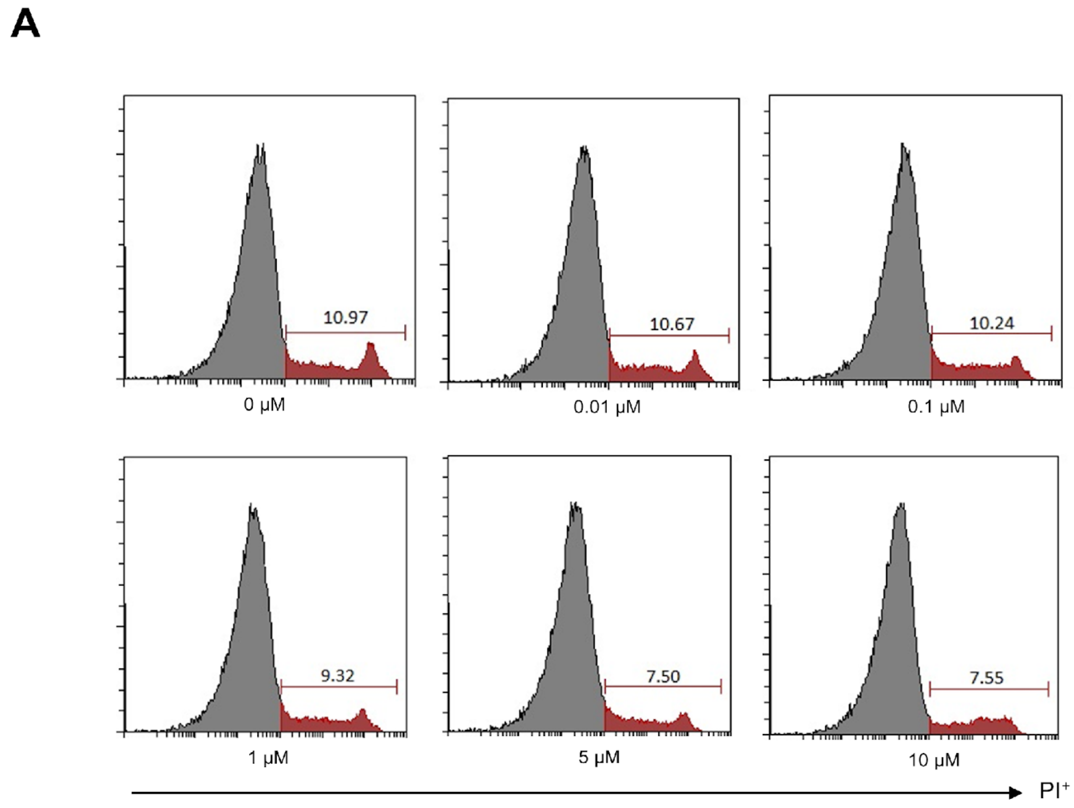

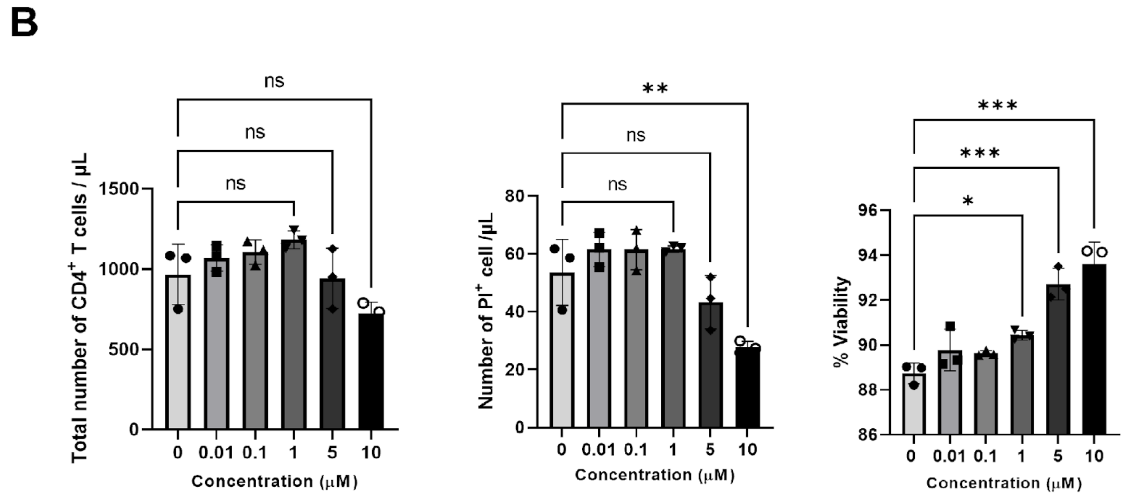

Supplementary Figure 3. Cell viability analysis. A and B, Human naïve $CD4^{+}T$ cells were stimulated with anti-CD3 and anti-CD28 and differentiated under Treg-polarizing conditions in the absence or presence of a selective DYRK1B inhibitor (AZ-DYRK1B-33) at 0.01, 0.1, 1, 5 and 10 μM for 96 h. Propidium iodide (PI) was used for cell viability staining. Histograms represent data from triplicated samples analyzed by flow cytometry and are summarized as bar graph. Data represent mean ±SD *$p < 0.05$, **$p < 0.01$, ***$p < 0.001$.

Supplementary Figure 4

A

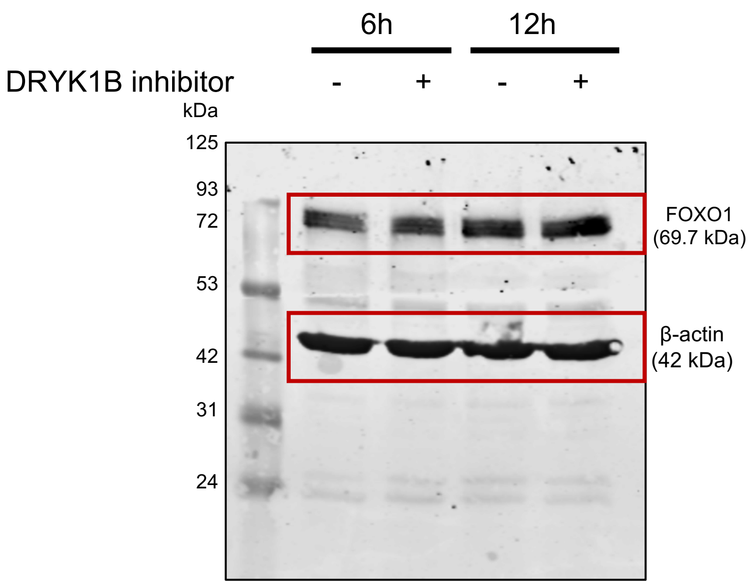

B

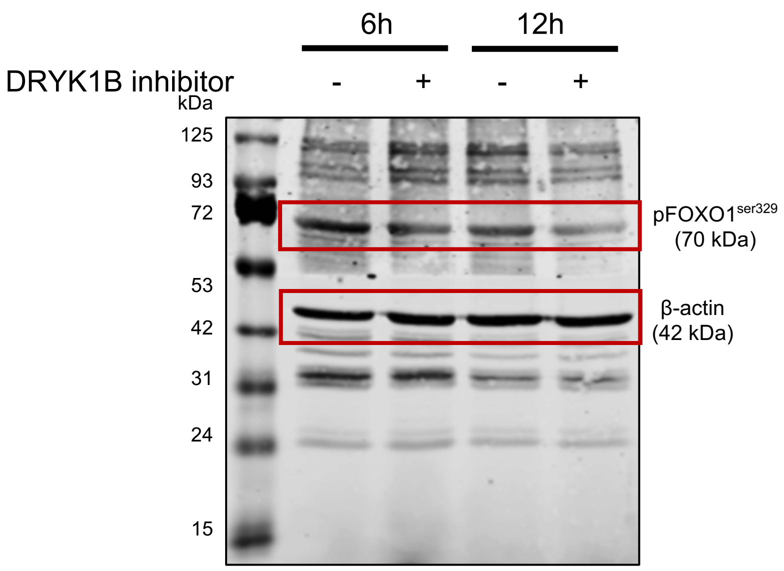

Supplementary Figure 4. A and B, A representative whole blot for western blot analysis of each protein in Figure 4A. Cells were stimulated with anti-CD3 and anti-CD28 and differentiated under Treg-polarizing conditions in the absence or presence of a selective DYRK1B inhibitor (AZ-DYRK1B-33) at 1 μM for 6 and 12 h. Total cell extracts were analyzed by western blotting with antibodies against the FOXO1, $pFOXO^{ser329}$ and β-actin.
